# A first-in-man, double blind, placebo controlled study of the candidate therapeutic vaccine Opal-HIV-Gag(c) in HIV infected patients receiving HAART

**DOI:** 10.1186/1742-4690-9-S2-O54

**Published:** 2012-09-13

**Authors:** AG Jackson, HN Kløverpris, A Handley, P Hayes, J Gilmour, M Atkins, B Walker, J Ackland, M Sullivan, P Goulder

**Affiliations:** 1 St. Stephen's AIDS Trust, London, UK

## Background

Preclinical studies of overlapping 15mer peptides spanning SIV, SHIV or HIV pulsed autologously ex vivo have demonstrated high level, virus-specific T cells responses and viral load suppression in Macaca nemestrina. The objective of this study was to evaluate the safety and preliminary immunogenicity of Clade C consensus peptides administered ex vivo to HIV positive adults.

## Methods

Synthetic 15mer peptides (n=123, Opal-HIV-Gag(c)) spanning Clade C, consensus Gag were manufactured to current good manufacturing practice and evaluated in a good laboratory practice toxicology study in Macaca mulatta. A first-in-human, single centre, placebo-controlled, double-blind, dose escalation study was conducted. Twenty three people with well controlled HIV (CD4+ > 350cells/mm3 and a HIV < 400 copies/mL), stratified by clade, were enrolled in four groups: 12mg (n=6), 24mg (n=7), 48mg (n=2) or matching placebo (n=8). Treatment was administered intravenously bedside (closed system) by enrichment of 120mL of whole blood for WBCs using a Sepax S-100 device, ex vivo mixing the peptides (or diluent alone) and incubation at 37°C for one hour prior to reinfusion. Subjects received 4 administrations at 4 weekly intervals followed by a 12 week post-treatment follow up. Immunogencity was assessed by ELIspot.

## Results

Opal-HIV-Gag(c) was generally well tolerated at doses of 12 and 24mg. There was an increased incidence of temporally associated pyrexia, chills, rigor, and transient/self-limiting lymphopenia in Opal-HIV-Gag(c) recipients compared to placebo. Only 2 subjects were recruited to the 48mg cohort. A serious adverse event of anuria, hypotension and tachycardia secondary to diarrhoea occurred following a single dose of vaccine at 48mg. No difference in ex vivo IFN-γ ELISpot response was observed at any time.

## Conclusion

An infectious cause for the event could not be identified, leaving the possibility of immunologically-mediated reaction to the vaccine thus leading to early termination of the study.

